# A minority of tuberculosis cases occurring during HIV care is possibly preventable

**DOI:** 10.1186/1758-2652-13-S4-P194

**Published:** 2010-11-08

**Authors:** L Skeie, T Skrede, A Maeland

**Affiliations:** 1 Oslo University Hospital Ullevaal, Dept. of Infectious Diseases, Oslo, Norway

## Background

It is often recommended to test patients presenting for HIV care for tuberculin skin test reactivity at the initial visit if they have a prior stay in tuberculosis-endemic areas.

## Purpose of the Study

To assess whether incident cases of tuberculosis (tb) in our cohort could have been prevented by such testing and subsequent preventive treatment of patients with a positive skin test.

## Setting

HIV clinic in northern Europe caring for about 1000 patients. During the observation time preventive treatment was only rarely prescribed.

## Methods

File review of all tb cases occurring more than two months after presenting for HIV care during the ten years 2000-2009. Cases were classified as possibly preventable, probably non-preventable or non-preventable based on residence and travel history, strain typing by restriction fragment length polymorphism technique and/or skin test reactivity.

## Results

24 cases in 23 patients were identified. 9 (38%) were classified as possibly preventable, 3 (13%) as probably non-preventable and 12 (50%) as non-preventable.

## Conclusion

A minority of tb cases occurring during HIV care is preventable by a policy of testing new patients from endemic areas and treating patients with positive skin tests.

